# Supplementary material for: Atroposelective Formal [2 + 5] Macrocyclization Synthesis for a Novel All-Hydrocarbon Cyclo[7] Meta-Benzene Macrocycle
Source: Molecules. 2024 Jul 17;29(14):3363. doi: 10.3390/molecules29143363 (PMC11279907; doi:10.3390/molecules29143363)
Supplement: Supplementary file 1 [file molecules-29-03363-s001.zip › molecules-3085688-supplementary.pdf]

Supporting Information

Atroposelective Formal [2+5] Macrocyclization Synthesis for a Novel All-hydrocarbon Cyclo[7] *m*-Benzene Macrocycle

Chao Gao,[a*] Hongchen Li,[b] Jing Zhao,[a] Lu-Lu Bu,[a] Mei Sun,[a] Jingrui Wang,[a] Gang Tao,[a] Longde Wang,[a] Li Li,[a] Gui-Lin Wen,[a*] and Yunhu Hu [a*]

a. School of Chemistry and Materials Engineering, Huainan Normal University, Huainan, 232038, P. R. China;

b. CNOOC Institute of Chemicals & Advanced Materials, Beijing 102209, China;

E-mail address: chaogao@mail.bnu.edu.cn, guilinwen@126.com, huyunhu@ustc.edc.cn.

Table of Contents

1. General Consideration S2

2. Synthesis of compounds S3

3. Single crystal X–ray structural of **CDMB-7** S5

4. References S7

5. NMR and HRMS Spectra S8

6. HPLC and Uv-vis for **CDMB-7** S16

1. General Consideration

All solvents, reagents and all deuterated solvents were purchased from commercial suppliers, including Innochem, Hwrk Chem, Energy Chemical and Cambridge Isotope Laboratories. Column chromatography was performed on silica gel (200–300 mesh) using a mixture of petroleum ether (PE; b.p. 60–90 ºC) / ethyl acetate (EA) as the eluent. All reactions are heated with oil bath (Note: Reaction solvents used are all analytical reagents (AR), and in-dividual special reagents have been marked) .

^{1}H and ^{13}C NMR spectra were recorded on Bruker AVANCE III 500WB，AVANCE III 400 and AVANCE 600 apparatus. ^{1}H and ^{13}C NMR chemical shifts are referenced to residual solvent signals (DMSO-d_6: δ H 2.49, δ C 39.6, chloroform-d ($CDCl_3$): δ H 7.26, δ C 77.16. ^{1}H NMR data are reported as follows: chemical shifts (δ ppm), multiplicity (s = singlet, d = doublet, t = triplet, q = quartet, m = multiplet, b = broad, ap = apparent), coupling constant (Hz), ^{13}C NMR date are reported in terms of chemical shift. High-resolution mass spectra (HMRS) were detected on Bruker Solarix XR FTMS spectrometer or AB SCIEX Triple TOF 5600+ apparatus. HMRS MALDI-FTICR were detected on Thermo LTQ-XL linear ion trap mass spectrometer. UV-vis spectra were measured on Shimadzu UV-2450. Melting points were measured on an SGW® X-4B apparatus and uncorrected. High performance liquid chromatography **(HPLC)** were measured on Shimadzu SCL-20AVP in in chromatographic column **AD-H** (Daicel).

Single crystals used to obtain the X–ray diffraction structures reported in the manuscript grew as colorless prism/plate, yellow plate, clear light yellow or gold prism. Diffraction grade crystals were obtained via slow evaporation using DCM/CH_3CN as the solvent. These crystals were analyzed by X–ray diffraction methods. The data were collected on XtalLAB Synergy or SuperNova, Cu at home/near, AtlasS2. Data reduction was performed using CrysAlisPro (Rigaku OD). The structures were refined by full–matrix least–squares on F2 with anisotropic displacement parameters for the non-H atoms using SHELXL-2014 or SHELXL-2018/3[1-3]. The hydrogen atoms were calculated in idealized positions with isotropic displacement parameters set to 1.2 x Ueq of the attached atom (1.5 x Ueq for methyl hydrogen atoms). Definitions used for calculating R(F), Rw(F2) and the goodness of fit, S, are given below and in the .cif documents. Neutral atom scattering factors and values used to calculate the linear absorption coefficient are from the International Tables for X-ray Crystallography (1992)[4]. All ellipsoid figures were generated using SHELXTL/PC[5]. Tables of positional and thermal parameters, bond lengths and angles, torsion angles, figures and lists of observed and calculated structure factors are located in the .cif documents available from the Cambridge Crystallographic Centre and may be obtained by quoting ref. numbers 2330139. The documents also contain details of the crystal data, data collection, and structure refinement for each structure.

2. Synthesis of compounds

2.1 General synthesis procedure of **1** (boronylated dimer): [6-7]

The commercial reagent 1,5-dibromo-2,4-dimethylbenzene **a** （100 g, 38 mmol, 0.038 M, 1 equiv.) was add to a 2L three-neck flask, and $CuCl_2$ (51.10 g, 0.38 mol, 10 equiv.) in a 100 mL solid sample injector. Under Ar, add 1 L of THF (Dry) to the three-neck flask to fully dissolve the solid sample. Place the reaction system in a -78 ℃ ice bath and cool for about 10 minutes. Place 143.00 mL of n-BuLi (5 mM) in a 250 mL constant pressure dropping funnel. When the reaction system temperature is stable, add it dropwise to the reaction system at a rate of 1 d/S (one drop per second). After the addition is complete, stir the reaction system at low temperature for an hour. Rotate the solid sample injector at this time, and add a small amount of $CuCl_2$ to the reaction system multiple times, then continue stirring for 12 hours. After the reaction is complete, add 100 mL of $NH_3 \cdot H_2O$ (12 M) to quench the n-BuLi in the reaction system. Remove THF by vacuum concentration, then add dilute HCl to dissolve the obtained solid. Wash with DCM (500 mL × 3) and H_2O (500 mL × 3). After vacuum concentrating the organic phase, perform column chromatography on silica gel (200-300 mesh) with eluent PE to obtain compound 5,5'-dibromo-2,2',4,4'-tetramethyl-1,1'-biphenyl (**b**, white solid, 6.95 g, 50%).

The reaction compound **b** (3.66g, 10 mmol, 0.067 M, 1equiv.) was add into a 500 mL three-necked flask, and pinacolboronate ester (B_2Pin_2, 3.81 g, 15 mmol, 0.10 M, 1.50 equiv.), followed by $Pd(dppf)_2Cl_2 \bullet CH_2Cl_2$ (154.0 mg, 0.19 mmol, 1.3×10^{-3} mM, 0.010 equiv.) and AcOK (7.84 g, 80 mmol, 0.53 M, 3.20 equiv.). Place in the reaction solvent 1,4-dioxane (150 mL). The reactiontemperature is 100 °C, and reaction time is 40 hours in Ar. After the reaction is complete, cool to room temperature. Remove dioxane by rotary evaporation, wash with DCM (150 mL × 3) and H_2O (150 mL × 3). Concentrate the organic phase under vacuum, and purify by silica gel (200-300 mesh) column chromatography using eluent EA to obtain compound **1** (white solid, 1.38 g, 30%).

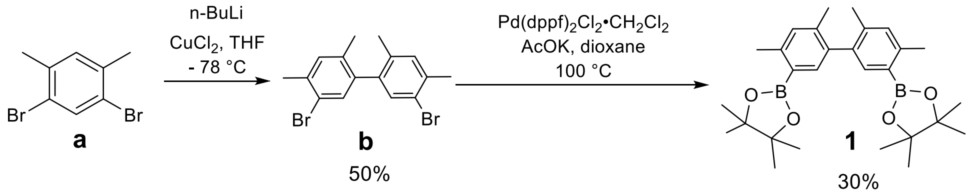

Fig. S1 Synthesis of **1**

2.2 General synthesis procedure of **2 (**dibrominated pentamer): [8]

According to the reported document, add **a** (50 g, 19 mmol, 0.095 M, 1 equiv.) to a 2 L three-necked flask, $Pd(dppf)_2Cl_2 \bullet CH_2Cl_2$ (154.00 mg, 0.19 mmol, 9.5×10^{-4} mM, 0.010 equiv.), AcOK (7.84 g, 80 mmol, 0.40 M, 3.20 equiv.), and B_2Pin_2 (7.24 g, 28.50 mmol, 0.14 M, 1.50 equiv.). Under Ar, the reaction temperature is 100 ℃, the solvent is dioxane (200 mL), and the reaction time is 30 hours. Then continue to add **a** (1.31 g, 5 mmol, 0.05 M, 1.00 equiv.), AcOK (3.45 g, 25 mmol, 0.25 M, 5.00 equiv.) to the reaction system, and finally add 100 mL of mixed solvent tetrahydrofuran/water (4/1, *v/v*). And the reaction temperature changes on 80 °C for 24 hours under Ar. After the reaction is completed,

cool to room temperature. After removing THF and dioxane by rotary evaporation, wash with DCM (50 mL × 3) and distilled water (50 mL × 3). After vacuum concentration, use EA as the eluent for silica gel (200-300 mesh) column chromatography to obtain **2** (white solid, 226.0 mg, 20%).

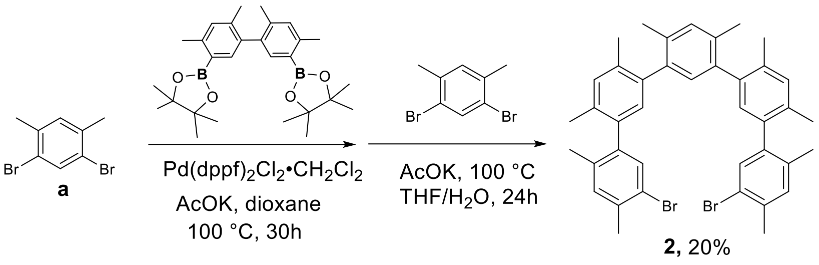

Fig. S2 Synthesis of **2**

2.3 Analytical data for compounds **b, 1, 2, CDMB-7:**

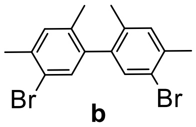

b

5,5'-Dibromo-2,2',4,4'-tetramethyl-1,1'-biphenyl (b): white solid (6.95 g, 50% yield), m. p. 80.1 - 80.3 ºC. ^{1}H NMR (500 MHz, $CDCl_3$, 273 K) δ 7.27 (s, 2H), 7.14 (s, 2H), 2.42 (s, 6H), 2.00 (s, 6H); ^{13}C NMR (125 MHz, $CDCl_3$, 273 K) δ (ppm): 119.4, 116.7, 115.1, 112.7, 112.2, 101.5, 57.0 (t, J = 32.5 Hz), 2.5, 0.8; ESI (*m*/*z*): $[M+H]^{+\bullet}$ calcd. for $C_{16}H_{17}Br_2$, 366.9692; found, 366.9690.

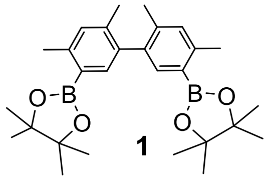

Boronylated dimer (1): white solid (1.38 g, 30% yield), m. p. 224.5 – 224.7 ºC. ^{1}H NMR (500 MHz, $CDCl_3$, 273 K) δ 7.52 (s, 2H), 7.08 (s, 2H), 2.57 (s, 6H), 2.05 (s, 6H), 1.34 (s, 24H); ^{13}C NMR (125 MHz, CDCl3, 273 K) δ (ppm): 123.4, 118.9, 118.0, 117.2, 111.2, 63.2, 57.0 (t, J = 31.2 Hz), 5.0, 4.8, 1.8, 0.0; ESI (*m*/*z*): $[M+H]^{+\bullet}$ calcd. for $C_{28}H_{41}B_2O_4$, 463.3186; found, 463.3193.

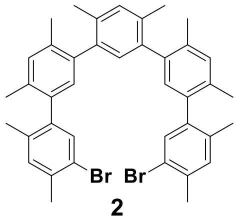

2

Dibrominated pentamer (2): white solid (226.0 mg, 20% yield), m. p. 262.1-262.7 ºC. ^{1}H NMR (400 MHz, $CDCl_3$, 273 K) δ 7.33 (d, J = 9.6 Hz, 2H), 7.18 – 7.12 (m, 5H), 6.95 – 6.91 (m, 1H), 6.87 (s, 2H), 2.42 – 2.40 (m, 6H), 2.14 – 2.01 (m, 24H).

Experimental procedure for Suzuki–Miyaura coupling "[2+5]" cyclization reactions: In a 25 mL Schlenk tube equipped with a stir bar were placed boronylated dimer **1** (0.15 mmol, 0.075 M, 1 equiv), dibrominated pentamer **2** (0.15 mmol, 0.075 M, 1 equiv), $Pd_2(dba)_3$ (5 mmol %, 3.75×10^{-3} M), and Cs_2CO_3 (0.75 mmol, 0.375 M, 5 equiv) in toluene (2.0 mL). The tube was evacuated and refilled with Ar three times. The reaction mixture was stirred at 100 °C for 12 h. After it was cooled, the reaction mixture was diluted with 10 mL of ethyl ether, and filtered through a pad of silica gel, followed by washing the pad of silica gel with the same solvent (20 mL). The filtrate was washed with water (3 × 15 mL). The organic phase was dried over Na_2SO_4, filtered, and concentrated under reduced pressure. The residue was then purified by flash chromatography on silica gel to provide the corresponding product.

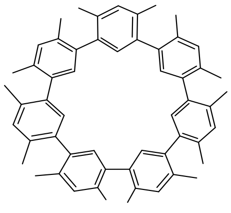

CDMB-7

Cyclo[7](1,3-(4,6-dimethyl)benzene) (CDMB-7): white solid (47.0 mg, 43% yield), ^{1}H NMR (500 MHz, TCE-d_2, 273 K) δ (ppm): 7.05 (s, 1H), 6.99 (d, J = 4.0 Hz, 4H), 6.95 (d, J = 5.5 Hz, 3H), 6.92 (s, 2H), 6.74 (s, 2H), 6.44 (s, 2H), 2.21-1.94 (m, 42H); ^{13}C NMR (125 MHz, TCE-d_2, 363 K) δ (ppm): 139.1, 139.0, 138.2, 138.8, 138.7, 138.5, 138.2, 137.9, 134.2, 134.4, 134.3, 133.2, 134.1, 133.6, 132.3, 131.8, 131.7, 131.3, 131.0, 130.9, 130.8, 130.4, 27.0, 19.9, 19.8, 19.8, 19.6, 19.5, 19.5; MALDI-FTICR HRMS (m/z): $[M]^{+\bullet}$ calcd. for $C_{56}H_{56}$, 728.43820; found, 728.43789.

3. Single crystal X–ray structural of CDMB-7

Take a 2.0 mL small glass bottle, dissolve 3.0 mg of **CDMB-7** in 2.0 mL of DCM. After the sample is completely dissolved, place the glass bottle completely in a glass bottle containing 4 mL of CH_3CN. Seal the bottle mouth with sealing film, then pierce 6-8 small holes with a fine needle, and let it evaporate at room temperature for 24 hours. At this time, colorless needle-like crystals will precipitate in the small glass bottle.

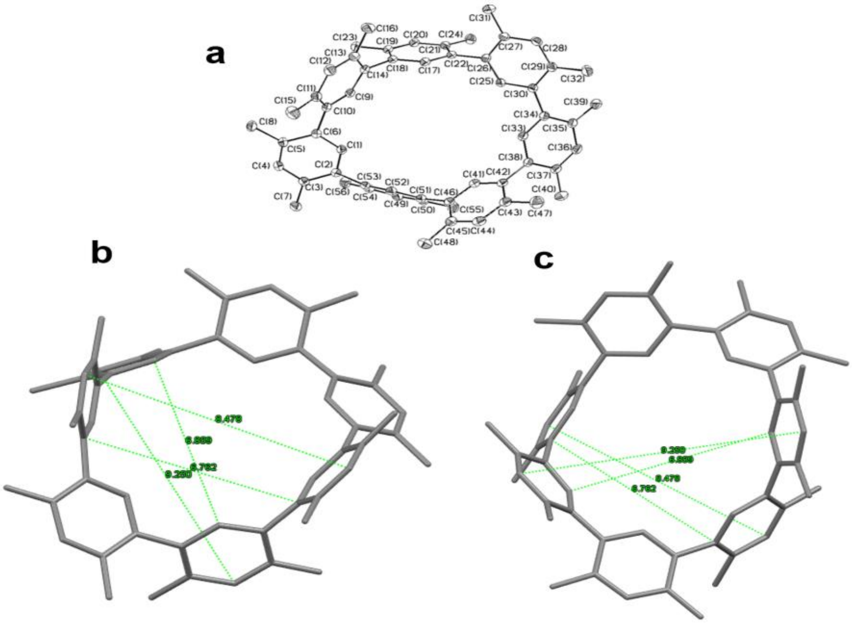

Fig. S3 (**a**) The single crystal structure of **CDMB-7**. Thermal ellipsoids is shown at 25% probability; (**b**) and (**c**) The diameter of **CDMB-7** in different views.

Table S1. Summary of single crystal X–ray diffraction analysis of **CDMB-7**

	**CDMB-7**
CCDC NO.	2330139
Description	block
color	colourless
From solution	CH_3CN / CH_2Cl_2
Empirical formula	C_{56} H_{56}
Mr	729.00
Crystal size (mm^3)	0.020×0.015×0.010
Crystal system	triclinic
Space group	P -1
a [Å]	13.4948(8)
b [Å]	13.7789(7)
c [Å]	14.3090(8)
α [deg]	66.604(5)
β [deg]	81.058(5)
γ[deg]	62.260(6)
V/ [Å3]	2159.8(2)
d/[g/cm3]	1.121
Z	2
T [K]	170K

R1, wR2 I > 2ó(I)	0.0649, 0.1025
R1, wR(all data)	0.1750, 0.2010
quality of fit	1.021

Single crystal X–ray diffraction features of **CDMB**-7 as follows:

Temperature: 170 K;

Absorption correction: Correction method= # Reported T Limits: T_{min}=0.820 T_{max}=1.000 AbsCorr = MULTI-SCAN

4. References

[1] Sheldrick, G. M. SHELXL97. *Program for the Refinement of Crystal Structures.* University of Gottingen, Gottingen, **1994**.

[2] Dolomanov, O. V.; Bourhis, L. J.; Gildea, R. J.; Howard J. A. K.; Puschmann, H. *J. Appl. Cryst.* **2009**, *42*, 339–341.

[3] Sheldrick, G. M. *Acta Cryst*. *A.* **2015**, *71*, 3-8.

[4] Wilson, A. *J. C. International Tables for X-ray Crystallography*. *Vol. C, Tables 4.2.6.8 and 6.1.1.4.* Kluwer Academic Press, **1992**.

[5] Sheldrick, G. M. *SHELXTL/PC (Version 5.03)*. Siemens Analytical X-ray Instruments, Inc., Wisconsin, **1994**.

[6] Dupau, P.; Renouard, T.; Bozec, H. L. *Tetrahedron Letters.* **1996**, *37*, 7503-7506.

[7] Yang, Y. D.; Gong, H. Y. W. *Chem. Commun.* **2019,** *55*, 3701-3704.

5. NMR and HRMS Spectra

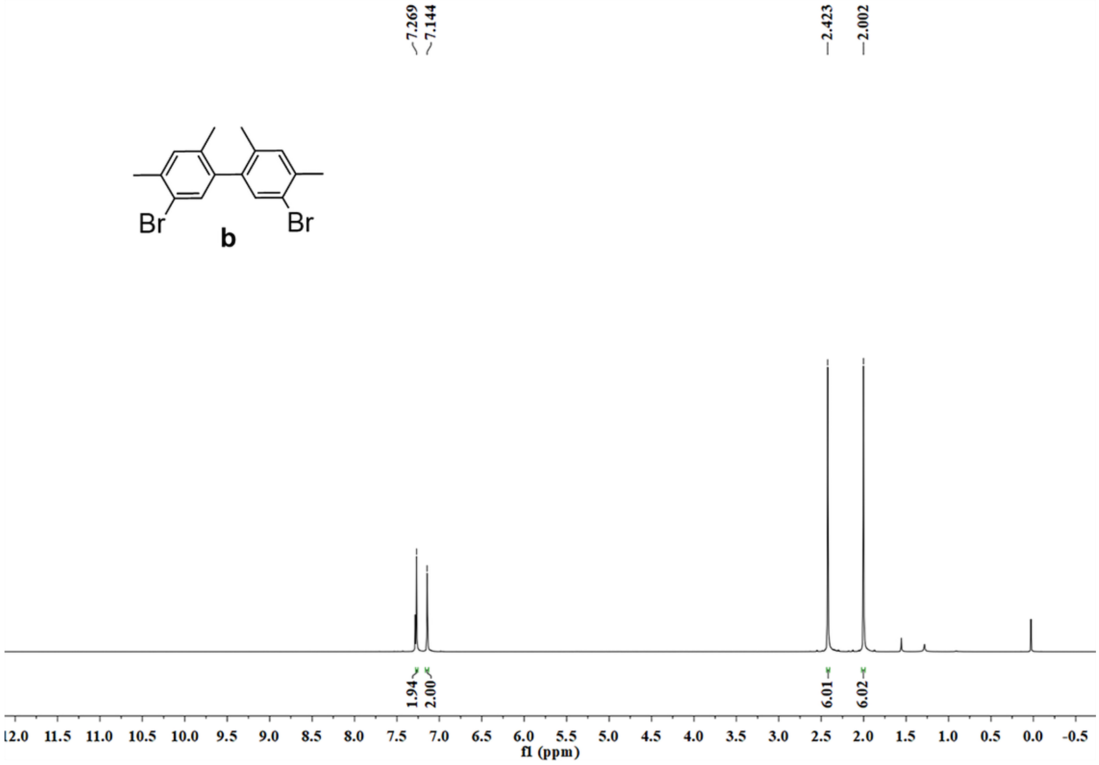

Fig. S4 The ^{1}H NMR spectrum of compound **b** (500 MHz, $CDCl_3$, 273 K)

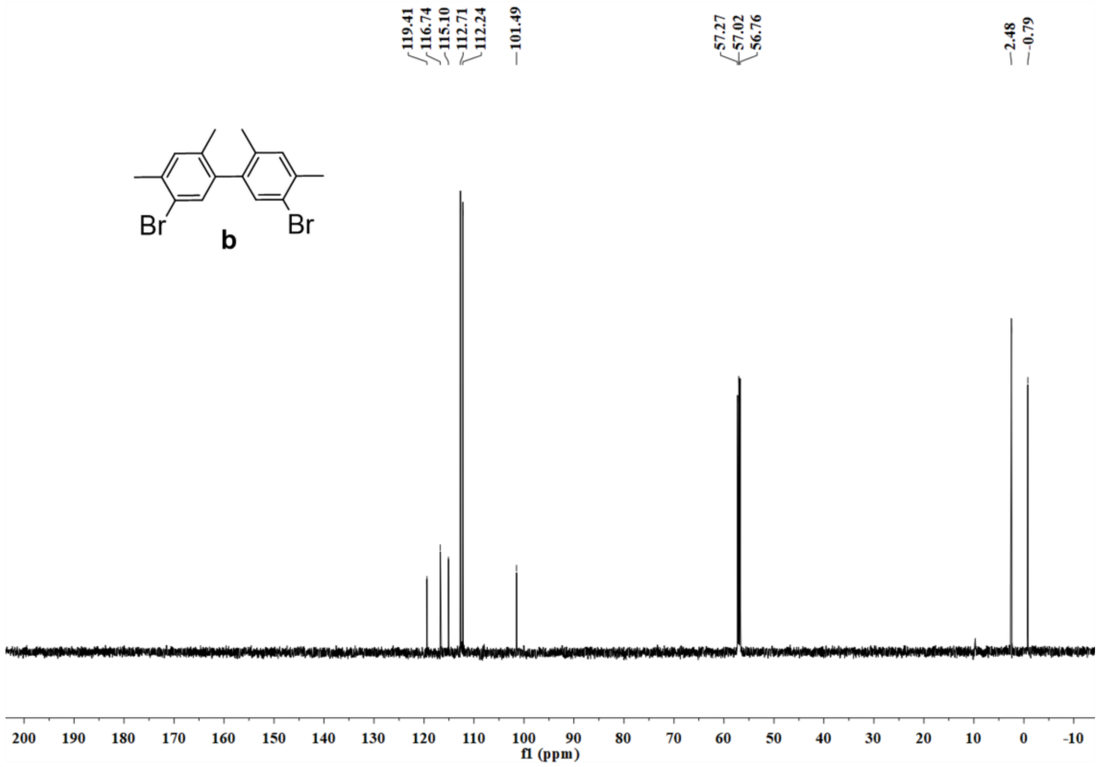

Fig. S5 The ^{13}C NMR spectrum of compound **b** (125 MHz, $CDCl_3$, 273 K)

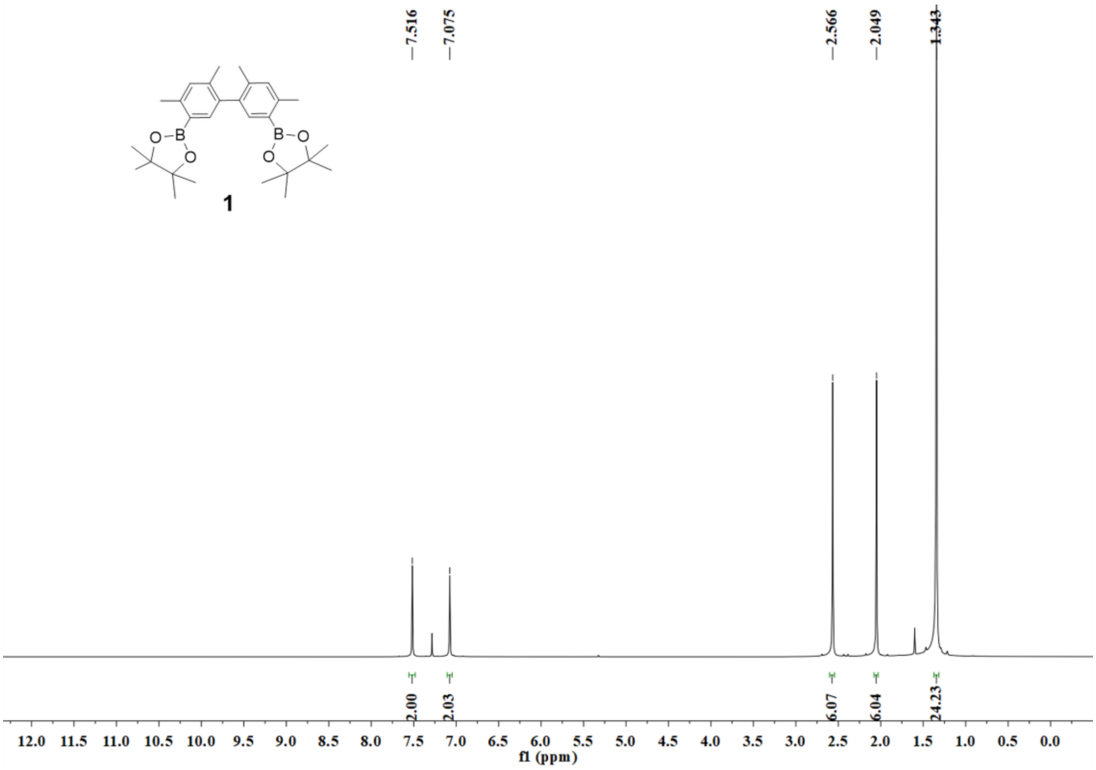

Fig. S6 The ^{1}H NMR spectrum of compound **1** (500 MHz, $CDCl_3$, 273 K)

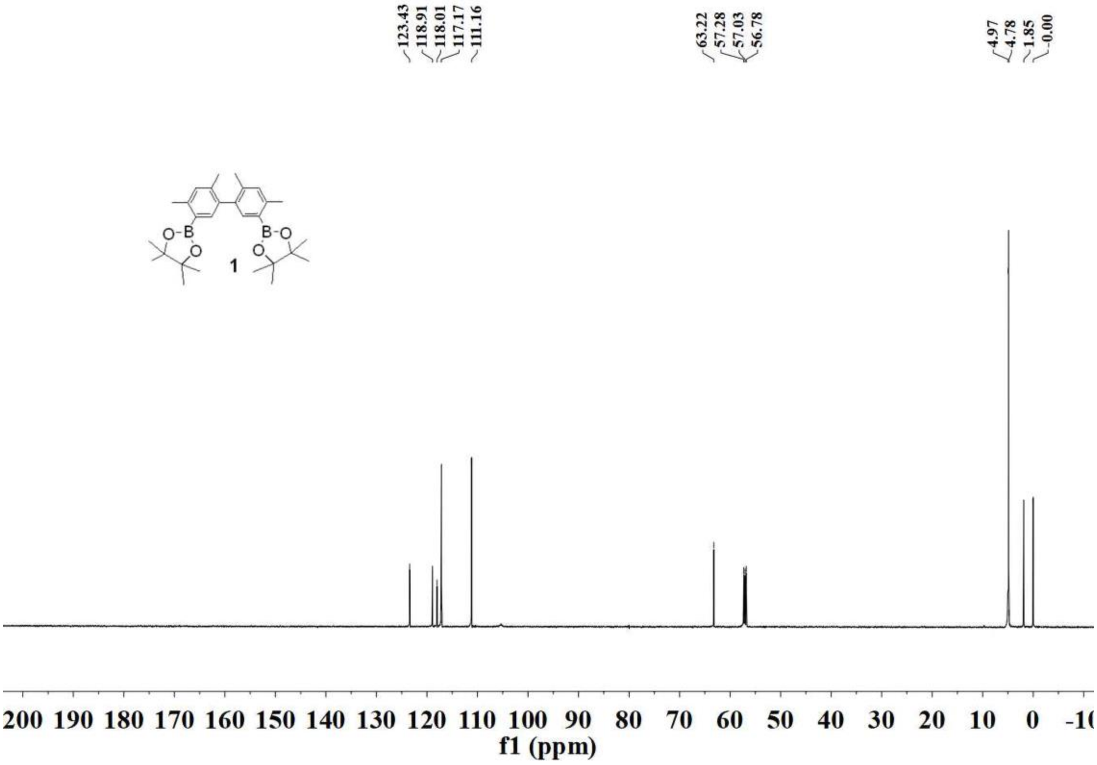

Fig. S7 The ^{13}C NMR spectrum of compound **1** (125 MHz, $CDCl_3$, 273 K)

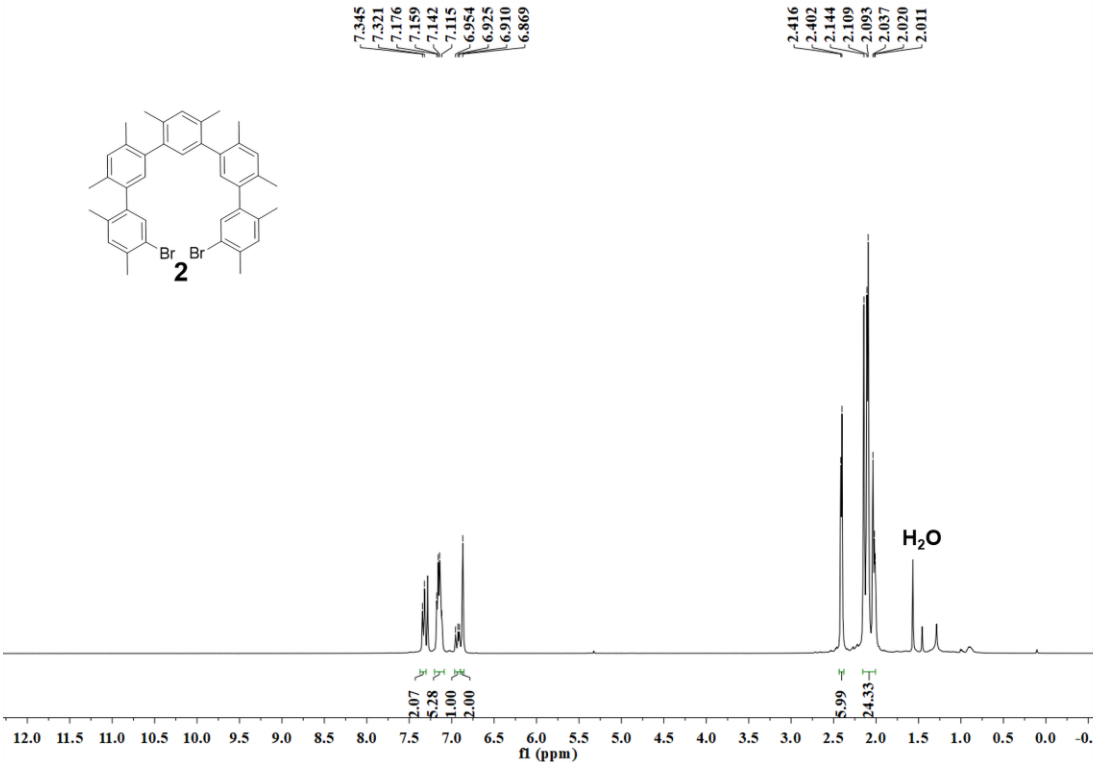

Fig. S8 The ^{1}H NMR spectrum of compound **2** (400 MHz, $CDCl_3$, 273 K)

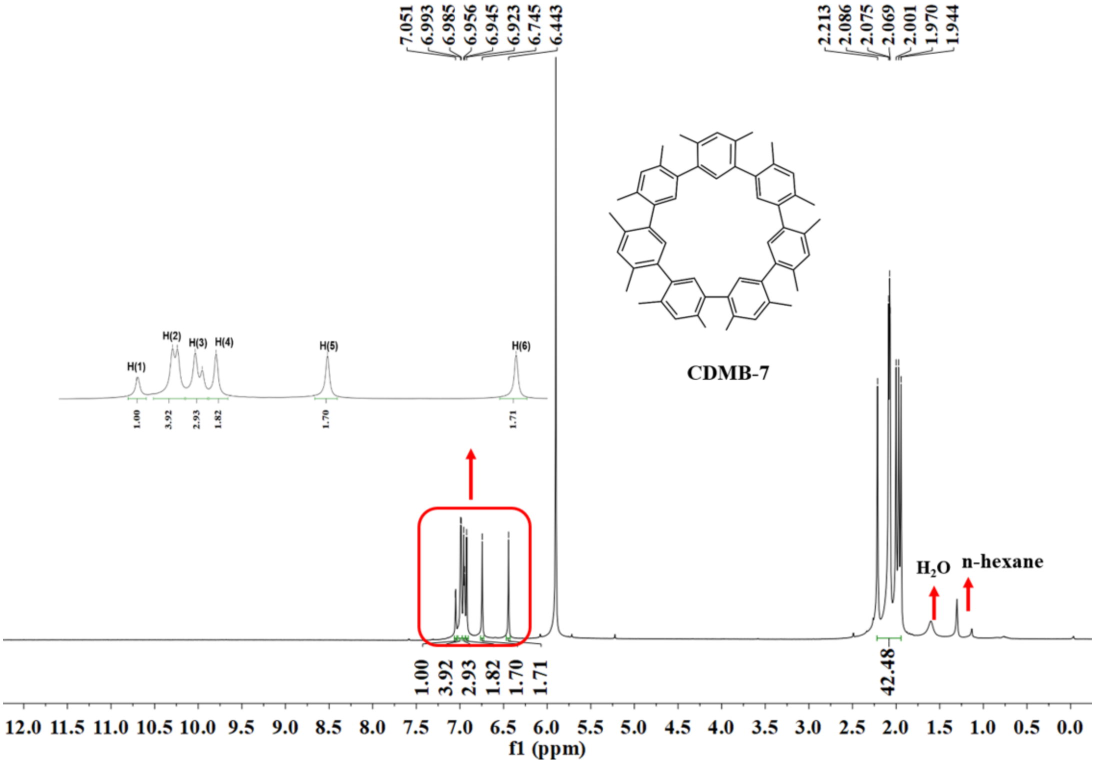

Fig. S9 The ^{1}H NMR spectrum of **CDMB-7** (500 MHz, TCE-d_2, 273 K)

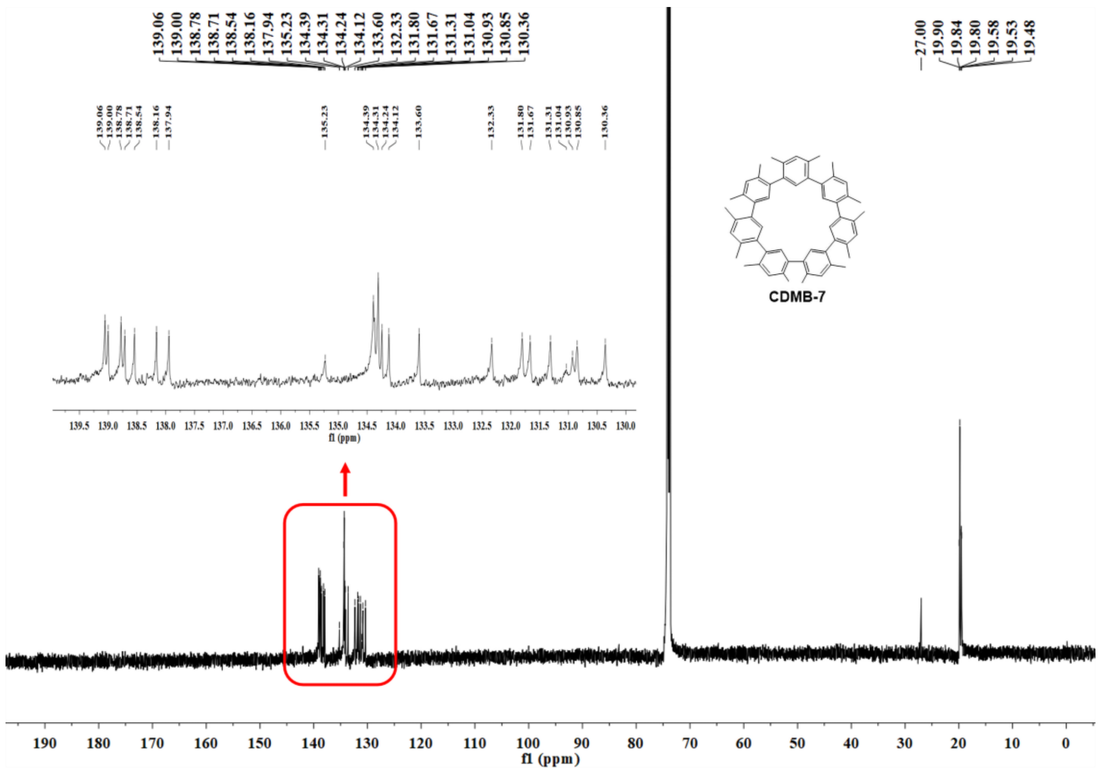

Fig. S10 The ^{13}C NMR spectrum of **CDMB-7** (125 MHz, TCE-d_2, 273 K)

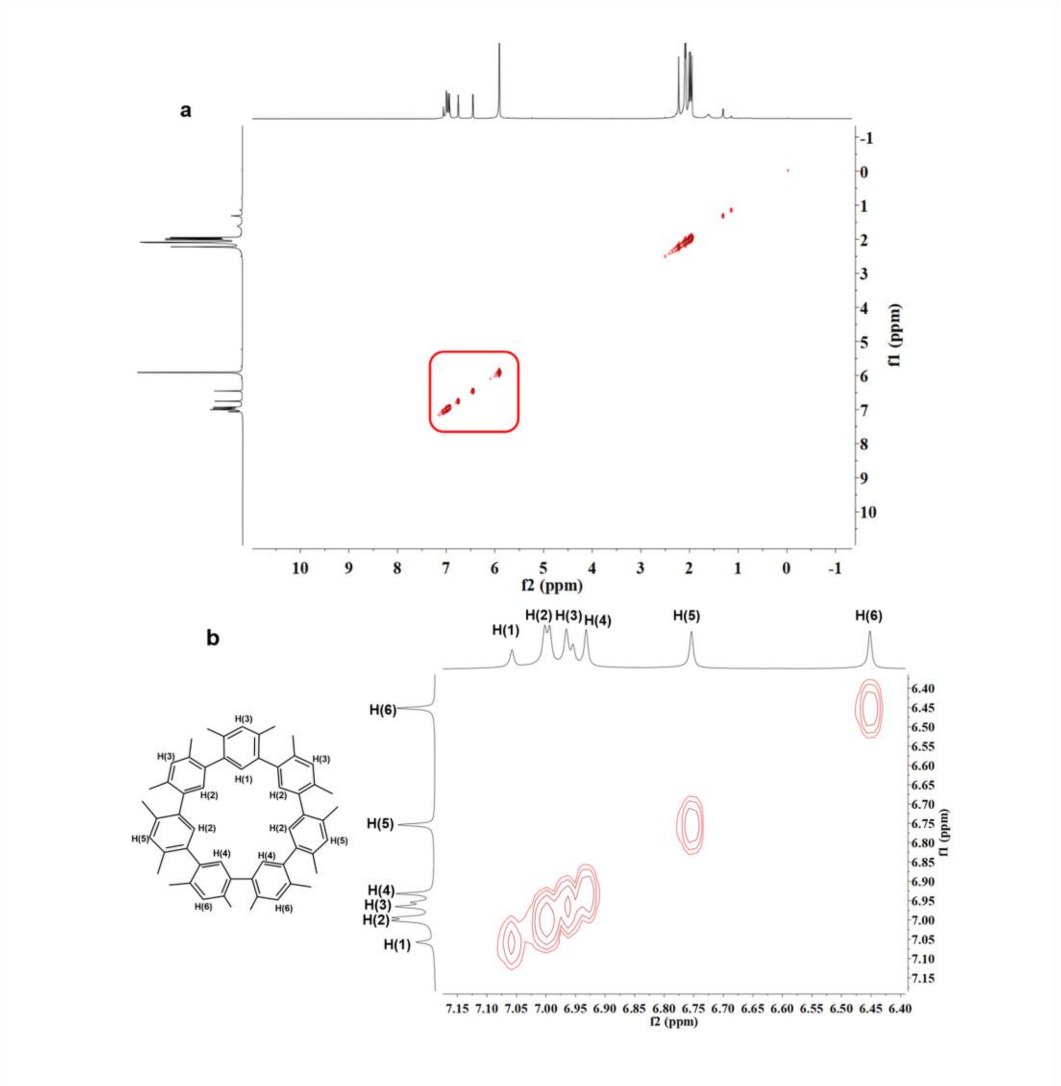

Fig. S11 Full view (a) and expanded view (b, red box annotation) of the COSY spectrum of **CDMB-7** (5.00 mM) in TCE-d_2 at 273 K (500 MHz).

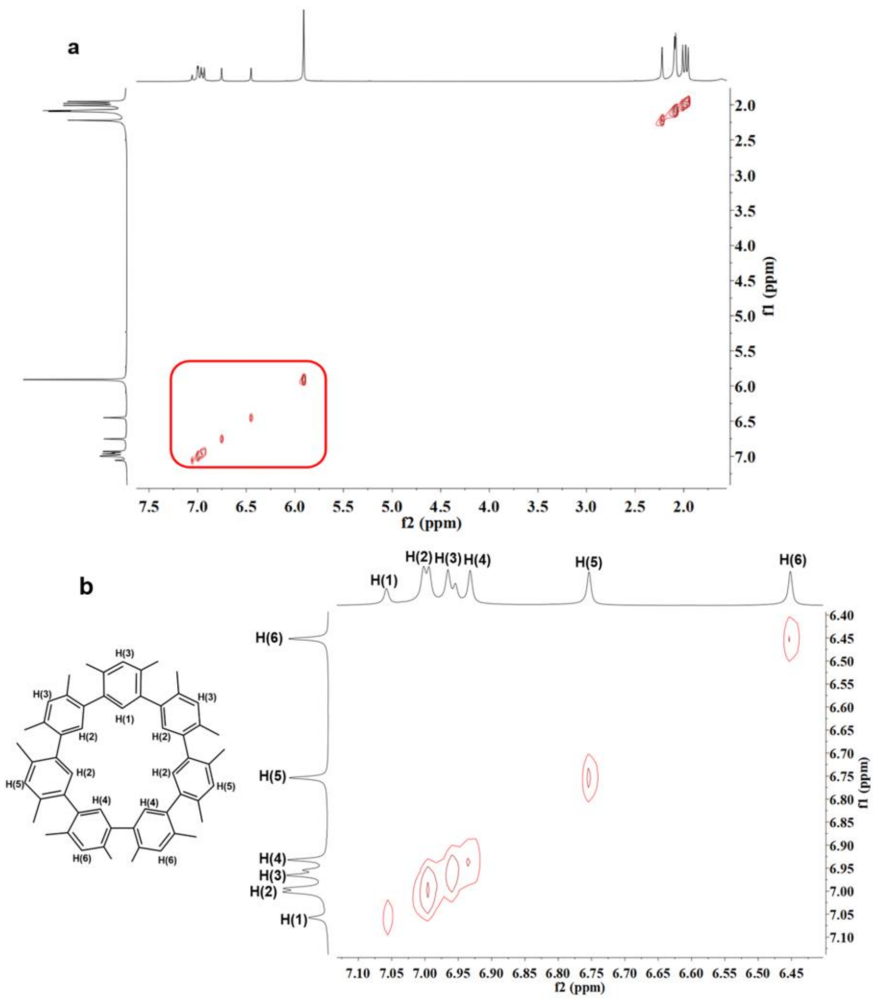

Fig. S12 Full view (a) and expanded view (b, red box annotation) of the NOESY spectrum of **CDMB-7** (5.00 mM) in TCE-d_2 at 273 K (500 MHz).

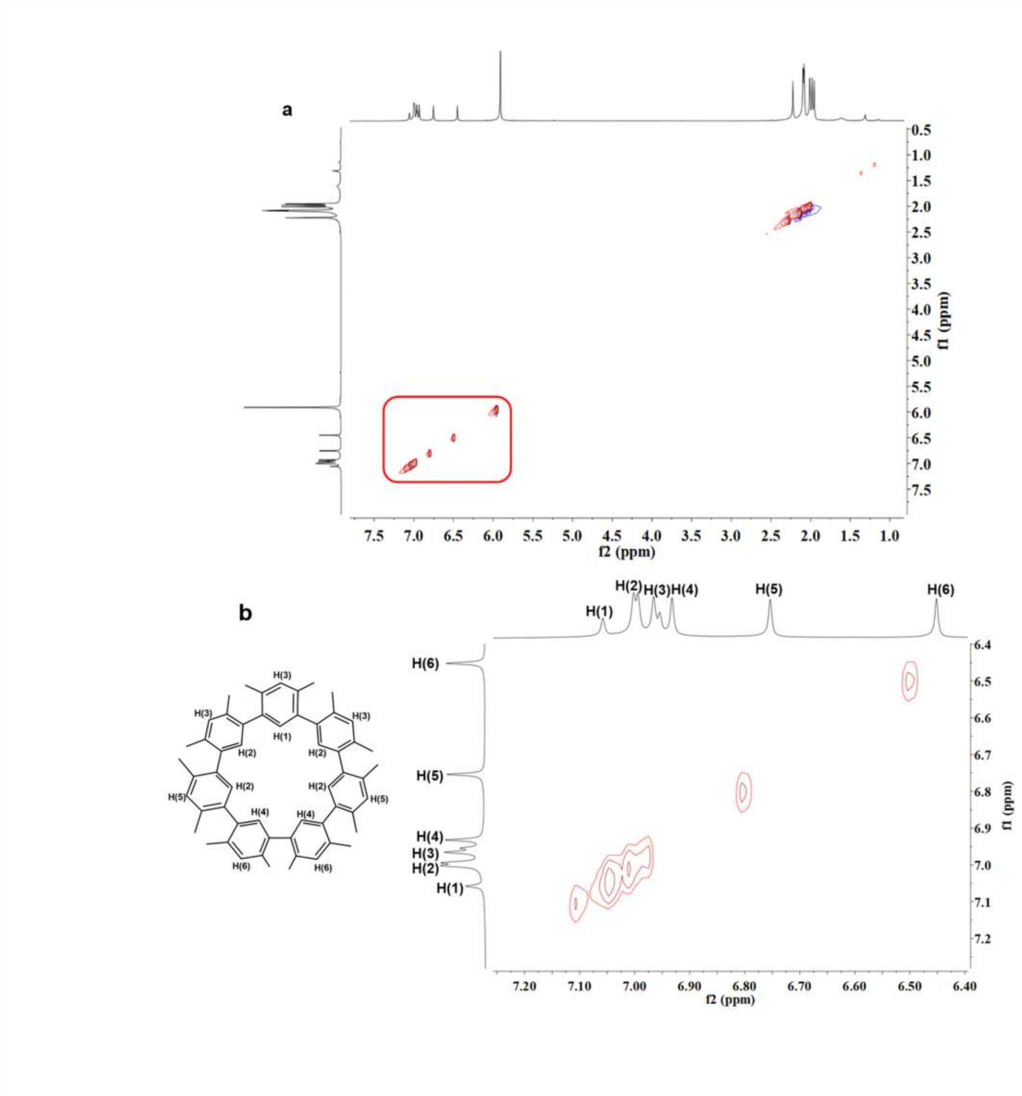

Fig. S13 Full view (a) and expanded view (b, red box annotation) of the ROESY spectrum of **CDMB-7** (5.00 mM) in TCE-d_2 at 273 K (500 MHz).

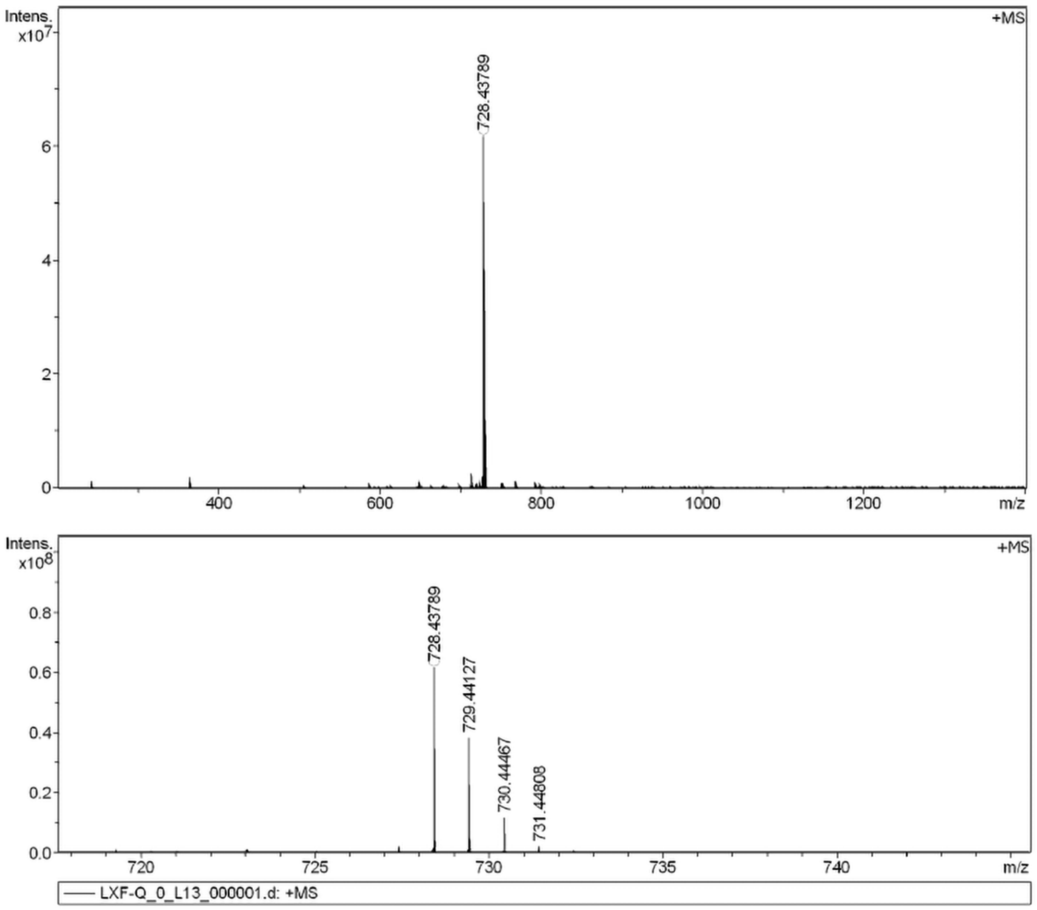

Meas. m/z	#	Ion Formula	Score	m/z	err [ppm]	Mean err [ppm]	mSigma	rdb	e⁻ Conf	N-Rule
728.437887	1	$C_{56}H_{56}$	100.00	728.437653	0.3	-0.3	3.4	29.0	odd	ok

Fig. S14 Positive MALDI-FTICR HRMS spectrum of **CDMB-7**

6. HPLC and Uv-vis for CDMB-7

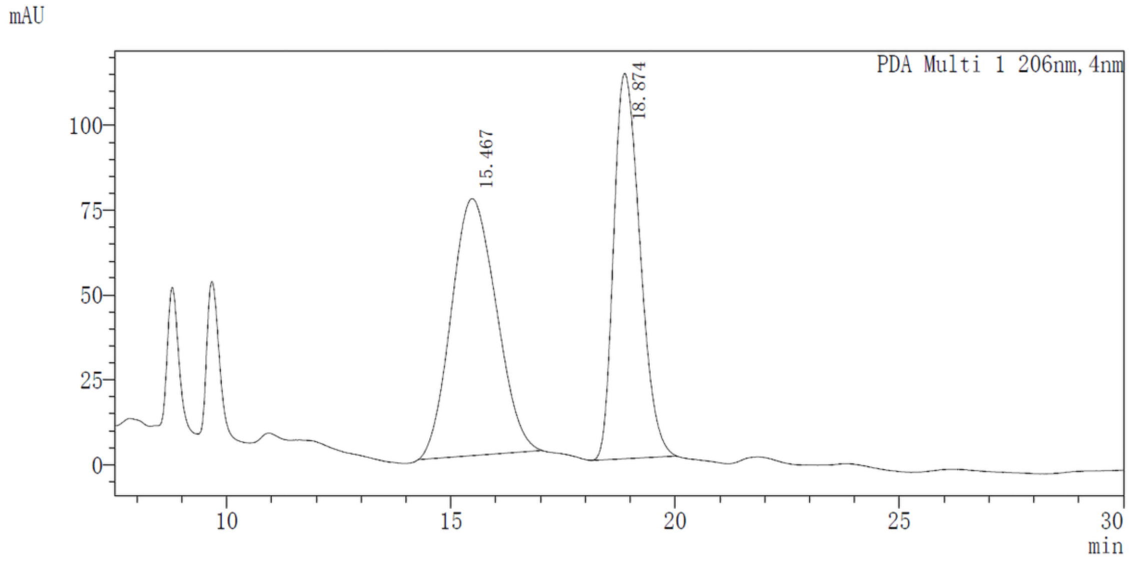

Fig. S15 HPLC full view spectrum of ***C*₂-CDMB-7** in chromatographic column **AD-H** (2 × 10^{-4} M, n-hexane/isopropanol, 95/5, *v/v*, 25 °C).

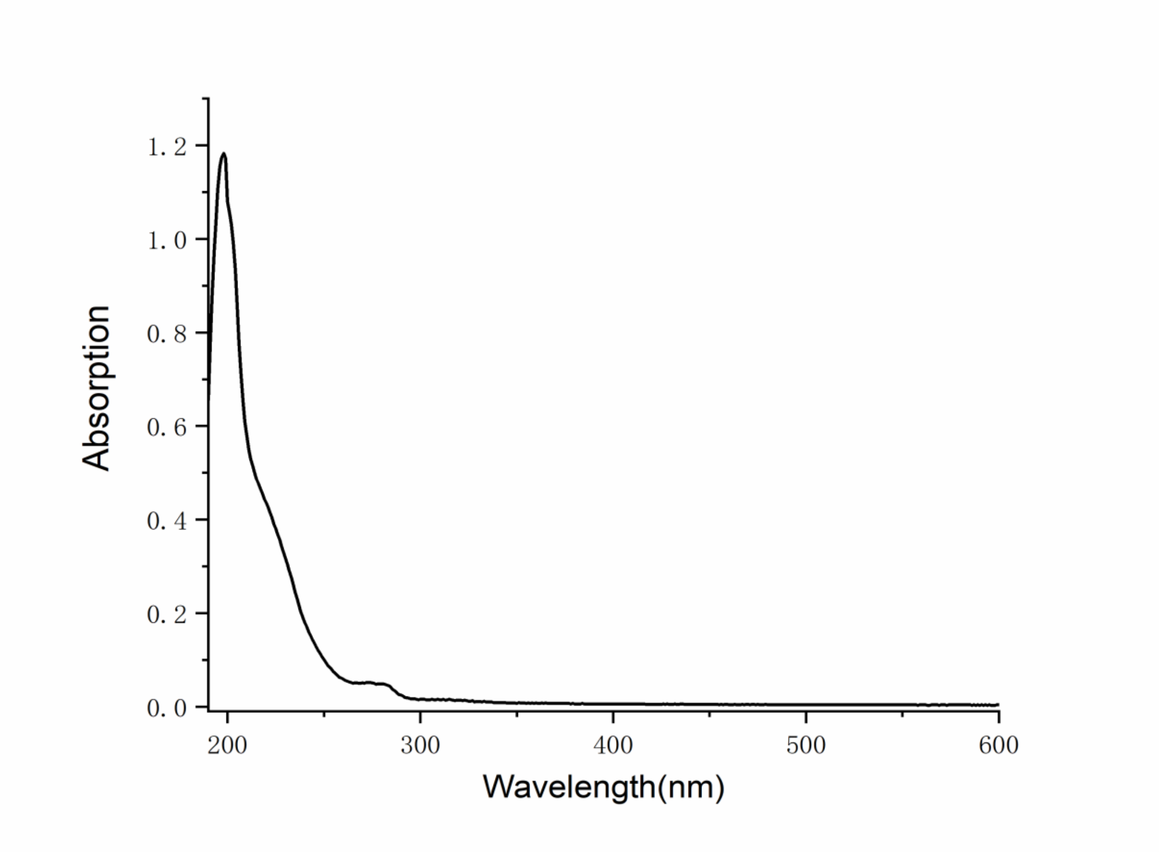

Fig. S16 Uv-vis f spectrum of ***C*₂-CDMB-7** (2 × 10^{-5} M, n-hexane, 25 °C).
